# Hutchinson-Gilford Progeria Syndrome: A Rare Genetic Disorder

**DOI:** 10.1155/2013/631378

**Published:** 2013-10-30

**Authors:** Rajat G. Panigrahi, Antarmayee Panigrahi, Poornima Vijayakumar, Priyadarshini Choudhury, Sanat K. Bhuyan, Ruchi Bhuyan, G. Maragathavalli, Abhishek Ranjan Pati

**Affiliations:** ^1^Department of Oral Medicine & Radiology, Institute of Dental Science, Siksha O Anusandhan University, K8 Kalinga Nagar, Bhubaneswar, Odisha 751003, India; ^2^Department of Pedodontics & Preventive Dentistry, JSS Dental College, JSS University, Sri Shivarathreeshwara Nagara, Mysore, Karnataka 570 015, India; ^3^Department of Pedodontics & Preventive Dentistry, Tamil Nadu Dr. M. G. R. Medical University, Ragas Dental College & Hospital, No. 191 East Coast Road, Uthandi, Chennai, Tamil Nadu 600096, India; ^4^Department of Oral Medicine & Radiology, Kalinga Institute of Dental Sciences, KIIT University, Bhubaneswar, Odisha 751024, India; ^5^Department of Oral Pathology, Institute of Dental Science, Siksha O Anusandhan University, K8 Kalinga Nagar, Bhubaneswar, Odisha 751003, India; ^6^Department of Oral Medicine & Radiology, Saveetha Dental College, Saveetha University, No. 162 Poonamalee High Road, Vellapanchavadi, Chennai, Tamil Nadu 600077, India

## Abstract

Hutchinson-Gilford progeria syndrome (HGPS) is a rare pediatric genetic syndrome with incidence of one per eight million live births. The disorder is characterised by premature aging, generally leading to death at approximately 13.4 years of age. This is a follow-up study of a 9-year-old male with clinical and radiographic features highly suggestive of HGPS and presented here with description of differential diagnosis and dental consideration. This is the first case report of HGPS which showed pectus carinatum structure of chest.

## 1. Introduction

Hutchinson-Gilford progeria syndrome (HGPS) is an extremely rare but devastating disorder characterised by dwarfism and premature aging [1]. It occurs sporadically with a reported incidence of one in eight millions and male predominance with M : F ratio of 1.5 : 1 and a strong racial susceptibility for Caucasians who represent 97% of patients [2]. The pattern of inheritance is uncertain, though both autosomal dominant and autosomal recessive modes have been proposed [3, 4]. Recent genetic advances have identified LMNA as a causative gene of HGPS. LMNA encodes laminins A and C, which are the main components of intermediate filamentous lamina, function as a structural support, and are essential for DNA replication and mRNA transcription [5]. Though the clinical presentation is typical, conventional radiological and biochemical investigations help in confirming the diagnosis. We present a case of progeria which showed classic physical and radiological changes of HGPS.

## 2. Case Report

**Figure 1:**
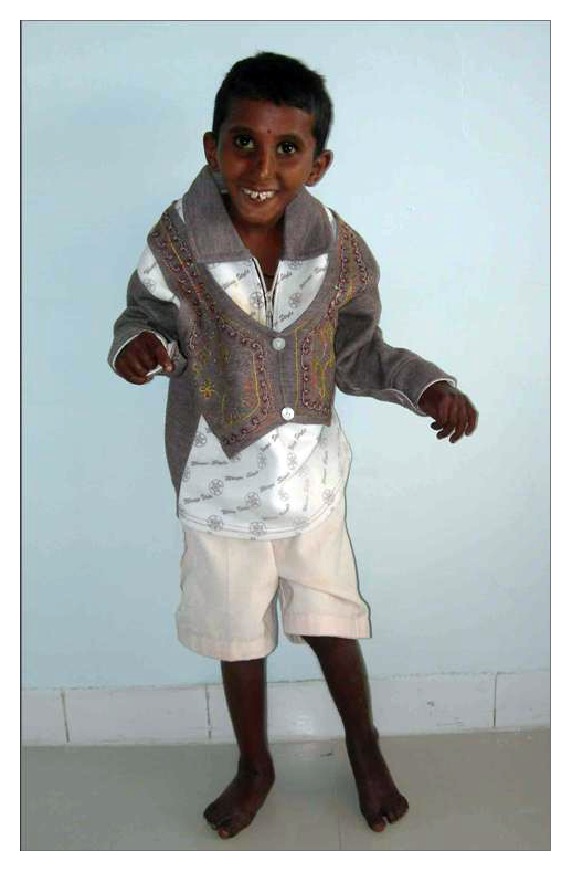
It shows a 9-year-old patient standing in an abnormal gait.

**Figure 2:**
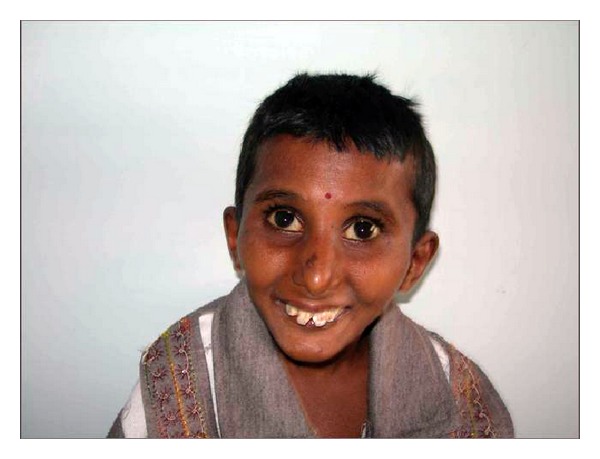
It shows the face of the 9-year-old boy showing typical features of progeria.

A 12-year-old male reported to the clinic with the chief complaint of decayed teeth in upper and lower anterior teeth region. Past medical history revealed that the first two years of his life were normal followed by failure to gain in both height and weight subsequently followed by loss of hair from scalp and eyebrows. Then he developed stretching of skin and inability to stand or walk properly; however, mental development was normal. Medical history revealed that the patient was undergoing treatment for acute hepatitis (see Figures 1 and 2).

**Figure 3:**
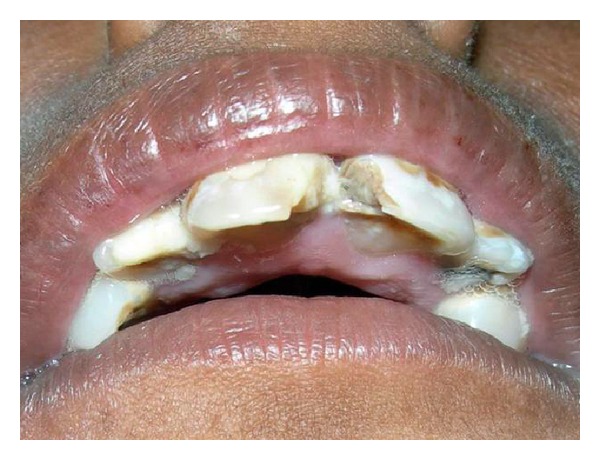
It shows the dental condition 2 years back.

**Figure 4:**
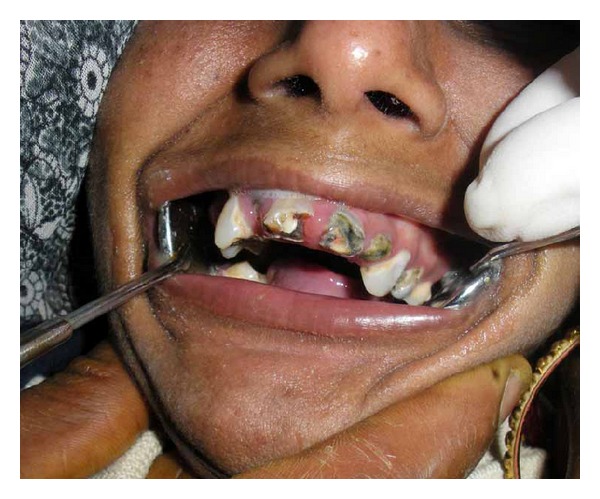
It shows the present dental condition.

Past dental history revealed that the patient came earlier to the hospital for the treatment of dental caries in the maxillary anterior 2 years back. But no treatment could be done as the patient was uncooperative. This second born child of a nonconsanguineous marriage had an uneventful prenatal history, with an other sibling being normal (see Figures 3 and 4).

No other family members were affected with similar complaints. On general examination, the young patient resembled a “wizened little old person.”

**Figure 5:**
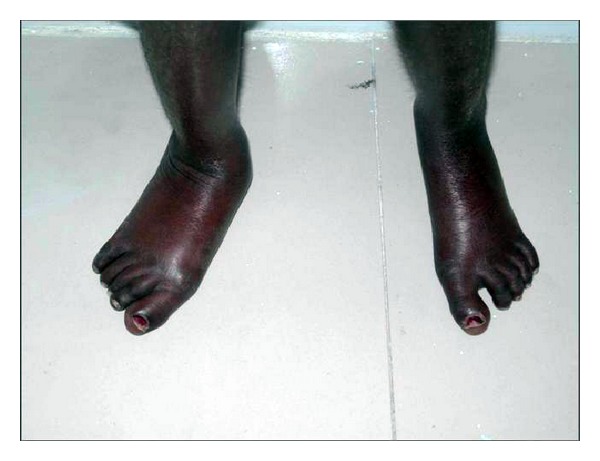
It shows the legs of the patient exhibiting shiny and dry skin.

**Figure 6:**
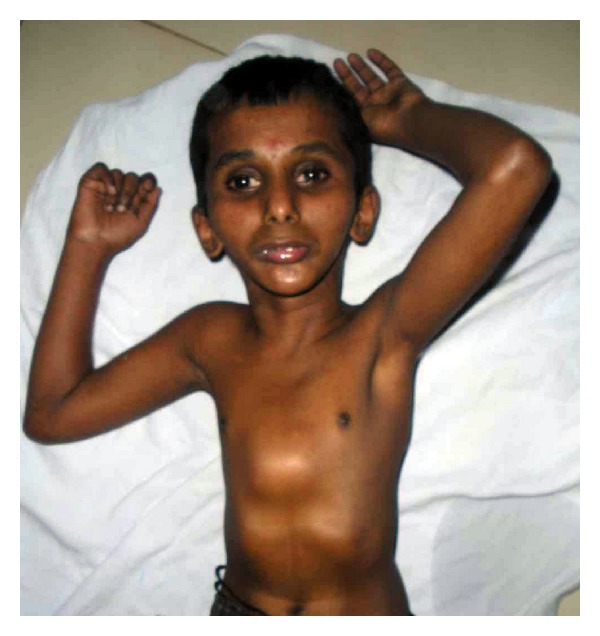
It shows the patient being unable to stand and is lying down, and chest showed pectus carinatum structure.

 He was thin built, poorly nourished, short statured with abnormal gait, thin atrophic skin, loss of subcutaneous fat around the extremities and the skin was coarse, stretched, shiny, and thickened. Chest revealed pectus carinatum structure (see Figures 5 and 6).

Systemic examination revealed that patient had impaired vision, slurring of speech, loss of memory, breathlessness, palpitation, and restricted joint movements with inability to stand or walk.

On extra oral examination, the patient had mild frontal bossing, a beaked nose, protruding eyes, a high pitched voice, and hypoplastic maxilla and mandible with mild mid facial deformity giving “plucked bird appearance.” Opening of mouth was restricted (interincisal distance-21 mm) and lateral movements of temporomandibular joint were also restricted.

On intraoral examination, the teeth were of normal size when compared to small size of the jaw, fractured crown irt to 11, root stump irt 12, 15, 16, 21, 22, and dental caries irt 13, 14, 23, 24, 25, 31, 32, 41, 42, 45. High arched palate and partial anodontia were seen. 

Based on history and clinical findings, a provisional diagnosis of progeria was made. To confirm the diagnosis, the child was subjected to radiological and biochemical investigations.

Biochemical investigations showed increased serum cholesterol which was 228 mg% and increased urinary excretion of hyaluronic acid. 

**Figure 7:**
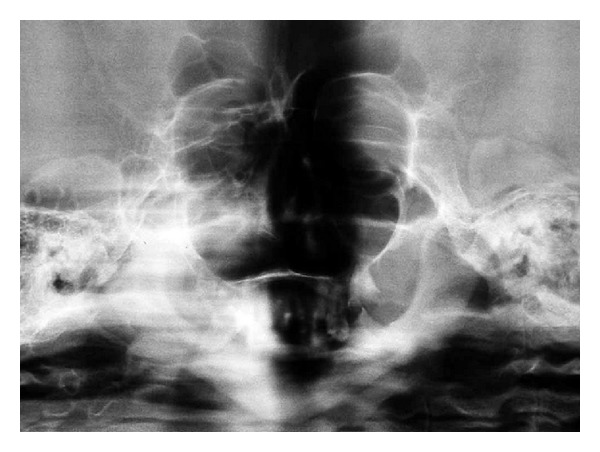
It shows the OPG with deformities in the mandible and partial anodontia.

OPG revealed hypoplastic maxilla, hypoplastic mandible with infantile angle, and multiple missing teeth. The morphology of the condyle appeared to be altered (see Figure 7).

Correlating the history, clinical features, radiographic findings, and laboratory investigations, the findings were consistent with HGP syndrome.

After careful systemic monitoring, extraction of the grossly decayed teeth was planned under antibiotic coverage.

## 3. Discussion

Progeria is a rare genetic disorder phenotypically characterised by feature of premature aging first described by Hutchinson in 1886 [6]. The term progeria was coined by Gilford in 1904 and is derived from the greek word “gerios” meaning old. DeBusk in 1972 renamed this condition as “Hutchinson-Gilford progeria syndrome” [2]. The rate of ageing in the affected individual is accelerated by seven times that of the normal. The average life span is 13 years (ranging from 7 to 27 years) with occasional survival till the age of 45 years. This is due to various abnormalities of mesodermal tissues and decreased survival time of fibroblasts. De novo mutation of LMNA which encodes for a major constituent of the inner membrane lamina has been reported [5]. Affected children are normal at birth and grow normally until about the end of the first year when both normal growth and weight gain slow down. 

The present case exhibited the typical phenotype of HGPS, showing the initial symptoms in the first year of life, severe growth deficiency, expressive lipodystrophy, loss of hair from scalp and eyebrows along with sclerodermatous changes giving rise to characterstic “Plucked Bird” appearance.

The scalp veins become prominent because of loss of subcutaneous fat and loss of hair. These patients are usually short and thin with an average height of 100 cm and average weight of 12–15 kgs or even less. Delayed dentition and hypodontia are also common [7].

The patient in our case had the classic features which included facial features, alopecia, failure to thrive, poor sexual maturation, and normal intelligence.

Skeletal survey reveals the following radiological features: calvarium is thin and relatively large and the diploic space is absent or very shallow. The face is small with disproportionate small mandible that retains its infantile obtuse angle and short ascending rami. The clavicles are small in caliber and rarefied at birth [8]. This case typically presented with the above mentioned radiographic features confirming the provisional diagnosis.

In progeria, hyperlipidemia is often present with increased low density lipoproteins and increased serum cholesterol level, as seen in our patient. There is also an increase in hyaluronic acid levels that is responsible for sclerodermatous changes and cardiovascular changes. 

Death is mainly due to cardiovascular complication like myocardial infarction or congestive cardiac failure. Failure to thrive in patients with progeria may be due to a bioinactive form of growth hormone and lack of vasculogenesis caused by excessive secretion of hyaluronic acid [9].

The differential diagnosis that can be thought of in these cases includes Werner syndrome, acrogeria, Rothmund-Thomson syndrome, and Cockayne syndrome [10].

There is no effective treatment to date. The only available approach towards symptomatic treatment and timely identification and prompt management of complications.
